# The influence of Social Media Use of older adults on the Exercise Habits: The chain mediating effects of active coping strategies and perceived social support

**DOI:** 10.1371/journal.pone.0348566

**Published:** 2026-05-11

**Authors:** Qiu Jie, Mengfen Liu, Jiawei Chen, Jianhua Zhang, Shaokai Tang, Zeng Zhou

**Affiliations:** 1 Hunan Mechanical and Electrical Polytechnic, Changsha, Hunan, China; 2 Hunan university of medicine Physical Education and Arts College, Huaihua, Hunan, China; 3 College of Sports Science, Jishou University, Jishou, Hunan, China; 4 Department of Physical Education, Central South University, Changsha City, Hunan Province, China; Pingdingshan University, CHINA

## Abstract

With the advancement of internet adaptation for the older adults, social media has increasingly become an important platform for older adults to access health information and maintain social connections. At the same time, older adults’ Social Media Use behavior has a unique impact on the development and maintenance of their Exercise Habits.

**Methods:**

To explore the mechanisms through which Social Media Use affects older adults’ Exercise Habits, a cross-sectional design was employed. A convenience sampling method was used to select 1,119 individuals aged 60 and above. Social Media Use, Exercise Habits, Active Coping Strategies, and Perceived Social Support Scales were used for the survey, and the mediating roles of Active Coping Strategies and Perceived Social Support in the relationship between the two were analyzed.

**Results:**

Social Media Use significantly positively influenced older adults’ Active Coping Strategies and Perceived Social Support. Furthermore, Active Coping Strategies and Perceived Social Support each had a significant positive impact on older adults’ Exercise Habits. Notably, Social Media Use exerted a significant positive indirect effect on Exercise Habits through the chain path of Active Coping Strategies → Perceived Social Support.

**Conclusion:**

The impact of Social Media Use on older adults’ Exercise Habits does not occur directly, but rather through a psychological chain, involving the acquisition of age-appropriate exercise resources, the formation of Active Coping Strategies, the perception of diverse social support, and the consolidation of Exercise Habits. This provides a theoretical basis and practical guidance for promoting healthy behaviors in older adults through social media interventions.

## Introduction

The 55th “Statistical Report on the Development of the Internet in China” indicates that the internet penetration rate in China has risen to 78.6%, with 1.108 billion internet users [1]. Social media has transformed from a tool beyond the digital divide to an important channel for older adults to integrate into society and manage their health. Although traditional views suggest that older adults’ exercise behaviors are more reliant on offline community organizations [2], some studies have revealed that Social Media Use may suppress physical exercise through the time substitution effect [3]. However, this explanation fails to account for the current surge in the number of older adultsusers of exercise-related apps [4]. By 2025, the revenue generated by exercise-related apps is expected to reach $13 billion, a 134% increase compared to 2020 [5].Unlike the younger population, older adults’ Social Media Use is predominantly practical in nature, and their Exercise Habits are more significantly constrained by physical abilities, social support, and coping strategies [6,7]. Existing research has confirmed the positive impact of social media on exercise behaviors in younger populations [8], yet there is a gap in studies focusing on the elderly. older adults’ exercise behaviors are more focused on maintaining health rather than improving physical appearance. How does Social Media Use align with their physiological characteristics and psychological needs? Does its impact differ from that observed in younger populations? Therefore, based on empirical research, this study focuses on examining the impact of Social Media Use on the Exercise Habits of older adults, providing insights and references for optimizing older adultscare services and promoting active aging.

Existing studies on the relationship between digital media use and older adults’ health behaviors have mainly adopted single theoretical frameworks, with limited integration of multiple theories and insufficient consideration of the specific characteristics of older adults.Regarding physiological characteristics, young and middle-aged populations are at the peak of physical functioning, and their exercise behaviors are typically oriented toward enhancing fitness and improving body composition [9]. Older adults face physiological limitations such as reduced muscle mass, impaired joint function, and a higher prevalence of chronic diseases [2], and their exercise behaviors must prioritize safety adaptation and chronic disease management, requiring low-intensity, targeted exercise guidance. In terms of psychological needs [10], exercise behaviors in young and middle-aged populations are largely driven by self-efficacy and intrinsic motivation. In contrast, the psychological needs of older adults are predominantly shaped by social connectedness and emotional belonging [11]. Their exercise behaviors exhibit a pronounced social dependency, necessitating psychological security through group identification and peer support [12]. Regarding digital usage behaviors, young and middle-aged populations employ social media in diverse and entertainment-oriented ways, encompassing information acquisition, social interaction, and entertainment consumption [13]. In contrast, older adults primarily use social media for practical purposes [7], mainly to access health information and maintain contact with family and friends, with relatively limited digital skills [14]. Regarding health behavior goals, young and middle-aged populations place a high demand on the immediacy and perceptibility of exercise outcomes [15]. For older adults, the core goals of exercise behaviors are maintaining basic physical fitness, preventing chronic diseases, and delaying aging [16], with a greater emphasis on sustainability and safety, and a longer perceptual timeframe for evaluating outcomes.Shen et al. combined social support theory and found that internet usage can enhance the positive effects of social support on physical activity among older adults [17], but the chain relationship between perceived social support mechanisms and psychological strategies was not elucidated. Nam et al. explained that social media improves the quality of life for older adults by accumulating social support, but the pathway of conversion from social resources to psychological resources was not distinguished [18]. Gyurcsik et al. validated the applicability of the Stress and Coping Model in physical activity among older adults, finding that cognitive appraisal of exercise-related stress and the selection of coping strategies directly influence exercise adherence [19]. However, these studies overlooked the compound effects of multiple digital environmental stimuli on stress appraisal and coping strategies. Ang et al. found that internet usage can enhance the quality of life for older adults by expanding their social support network, but the conversion of social resources into psychological resources was not addressed [20]. These studies all lack an in-depth exploration of the mechanisms underlying resource type conversion in the digital age.Based on the characteristics of older adults, this study integrates the Stress and Coping Model, social support theory, and resource conservation theory to construct an integrated framework: digital environmental stimuli → resource accumulation → psychological strategy activation → health behavior output. Compared to existing studies, this research clarifies the chain transmission mechanism from external social resources to internal psychological resources and health behaviors, addressing the gap in prior research that overlooked the interaction between social support and coping strategies. It expands the application boundary of core theories in the digital age, validates the applicability of this integrated theoretical framework in the field of digital health behavior among older adults, and provides a new theoretical perspective for related research.

## 1 Literature review

### 1.1 Social media use of older adults and exercise habits

Although a substantial body of research on Social Media Users has been accumulated both domestically and internationally, the majority of these studies have primarily focused on university student populations [21], and systematic exploration of the Social Media Use of Older Adults behavior remains relatively scarce.Social media, as a virtual social space, is characterized by its core features of interactivity, publicity, and community attributes [13,22].The behavior of Social Media Use of Older Adults can be divided into three core dimensions: information acquisition, social motivation, and behavioral modeling [7].First, exercise behavior among older adults is often constrained by practical barriers, such as insufficient health knowledge and a lack of awareness regarding appropriate exercise methods [23].Social media, relying on its convenience and precision in information dissemination, provides older adults with low-cost, accessible exercise guidance resources.Research by Helbostad et al.[24]indicates that over 60% of adults aged 60 and above, after obtaining health knowledge through Mobile Health Applications, actively adjust their lifestyles, including increasing the frequency of physical exercise.Additional studies confirm that the intensity of Social Media Use is significantly positively correlated with the physical activity levels of older adults [25], with the ease of information acquisition serving as the core driving factor of this relationship.Second, exercise behavior among older adults is notably socially dependent [12], and social media, through its online-offline interactive model, constructs an exercise support network that transcends time and space limitations.On the one hand, older adults can receive positive feedback and recognition from family, friends, and peers through means such as WeChat Sports check-ins and fitness group interactions, thereby enhancing the emotional rewards of exercise behavior. On the other hand, social media fosters exercise-interest groups that, through mechanisms such as peer supervision and group challenges, increase the persistence of exercise behavior.Shen et al.’s study [[26]points out that internet use strengthens the positive effect of social support on physical activity in older adults; as the level of digital integration increases, the facilitative role of social support in exercise behavior becomes more pronounced.Third, the role model effect of senior fitness influencers on social media provides older adults with perceptible and replicable exercise examples.Compared to traditional media, social media possesses stronger interactivity and authenticity; exercise examples from peers not only effectively challenge the traditional belief that aging should be associated with inactivity but also significantly enhance the self-efficacy of older adults.Previous research has confirmed that peer influence within social networks significantly increases the likelihood of exercise participation among older adults— the higher the support level from a peer network, the greater the probability of engaging in physical exercise, with social media serving as the core medium that amplifies this peer effect [11,27].Based on the above theoretical and empirical analysis, the present study proposes Hypothesis 1: Social Media Use of older adults is significantly positively associated with their Exercise Habits.

### 1.2 The mediating role of active coping strategies

Active Coping Strategies are defined as an individual’s psychological tendency to actively adjust cognition and seek adaptive solutions in response to stress, serving as a core psychological mediator linking environmental stimuli and health behaviors [28]. Older adults experience multiple stressors during aging, including functional decline and the accumulation of health risks, while Social Media Use can positively shape their active coping tendencies through psychological empowerment pathways, thereby promoting the establishment and maintenance of Exercise Habits. The age-friendly promotion and widespread adoption of digital technologies provide older adults with novel avenues for psychological adaptation, with social media driving the development and optimization of Active Coping Strategies through a sequential pathway of resource provision → psychological empowerment → coping enhancement.First, exercise-related targeted information on social media provides older adults with concrete solutions to health-related stress, attenuating negative cognitions of helplessness and reinforcing positive beliefs in problem-solving ability [29]. For example, senior creators on short-video platforms such as Douyin share ‘silver fitness’ cases that not only visually demonstrate the feasibility of exercise but also convey a proactive approach to aging, subtly enhancing cognitive adjustment abilities among older adults.Second, the social interaction features of social media help older adults overcome geographical constraints, allowing them to receive emotional support through participation in fitness community interactions and online exercise check-ins, thereby alleviating feelings of loneliness and helplessness and reinforcing proactive help-seeking and problem-solving ability. When older adults encounter difficulties during exercise, they can rapidly obtain targeted advice through online social communities, and such immediate support gradually consolidates into a pattern of active coping behavior. Previous studies have demonstrated that the use of digital media can significantly enhance psychological resilience and positive affect among older adults, which constitutes the core psychological foundation of Active Coping Strategies [18,30].Exercise Habits essentially represent proactive behavioral strategies through which older adults cope with aging-related stress, while Active Coping Strategies facilitate the effective implementation of these behaviors via a progressive pathway of motivation activation → behavioral execution → persistence reinforcement. At the level of motivation activation, older adults with active coping tendencies are more likely to perceive exercise as a proactive solution to maintain health and slow aging rather than a passively undertaken task, and this intrinsic motivation effectively enhances the initiative of exercise behavior [16,31].At the behavioral execution level, the problem-solving ability within Active Coping Strategies helps older adults overcome practical obstacles in exercise. For instance, low-intensity, gentle exercises such as Tai Chi and Baduanjin can be chosen for joint pain, while fragmented schedules can be addressed through multiple short sessions, thereby preventing interruptions in exercise due to encountered difficulties. At the persistence reinforcement level, the cognitive adjustment ability inherent in Active Coping Strategies assists older adults in reconstructing their cognitive framework regarding exercise [32], transforming the negative perception of exercise as a burden into a positive perception of self-care and viewing occasional interruptions as flexible adjustments rather than failures, thereby significantly enhancing the stability of Exercise Habits [33]. Moreover, coping planning plays a critical mediating role in translating exercise intentions into actual behaviors, effectively bridging the intention-behavior gap [15].In summary, Social Media Use positively shapes older adults’ Active Coping Strategies through informational empowerment and social support, and Active Coping Strategies further facilitate the formation and consolidation of Exercise Habits. Based on the above theoretical and empirical analysis, the present study proposes Hypothesis 2: Active Coping Strategies play a significant mediating role in the relationship between Social Media Use of older adults and their Exercise Habits.

### 1.3 The mediating role of perceived social support

Perceived Social Support is defined as the care, recognition, and resource provision perceived by individuals from others or groups. It serves as a core socio-psychological protective resource for older adults to cope with aging-related stress and maintain healthy behaviors [34]. Social media, by reconstructing the social networks and support acquisition pathways for older adults, significantly enhances their Perceived Social Support levels, thereby providing sustained motivation for the establishment and maintenance of Exercise Habits. Offline social interactions for older adults are often limited by physical decline and reduced mobility. In contrast, social media, through a chain of processes including trans-spatial connectivity, group formation based on shared interests, and enhanced emotional interactions, effectively compensates for the structural deficiencies of traditional social support, thus significantly improving their Perceived Social Support levels. In the dimension of emotional support, social media provides older adults with efficient and convenient pathways for emotional expression and response. Through sharing exercise routines on WeChat Moments or interacting in Douyin comment sections, older adults can receive attention, encouragement, and empathy from friends, family, and peers. This immediate and personalized emotional feedback significantly strengthens their core perception of being cared for and needed [35,36]. For example, older adultsusers who share physical improvements after exercise in fitness groups receive positive feedback such as “Great job, keep it up!” or “Learning from you!” This feedback further reinforces their deep understanding of emotional support. In the dimension of instrumental support, the community interaction features of social media provide older adults with targeted, practical support related to exercise. When older adults face dilemmas such as selecting exercise equipment or creating workout plans, they can quickly obtain experience-sharing and specific advice from online fitness communities. This targeted instrumental support enables older adults to clearly perceive the objective willingness of others to provide practical assistance [37].In terms of information support, the dissemination of exercise-related knowledge and the exchange of experiences on social media essentially represent a form of implicit social support.Older adults, by browsing exercise tutorials and engaging in health-related discussions, can sense the presence of a community that shares a common concern for health issues. This resonance and support at the informational level further expand and deepen the core connotations of Perceived Social Support [18,38].Empirical studies have confirmed that the frequency of Social Media Use among older adults is significantly positively correlated with their perceived level of social support. In-depth participation in digital social activities can effectively expand their social support networks, enhancing the intensity, breadth, and validity of support perception [39,40].Based on Social Support Theory [41], Perceived Social Support provides systematic protection for older adults’ Exercise Habits through a multidimensional path involving motivation reinforcement, behavioral buffering, and persistence enhancement.At the level of motivation reinforcement, older adults who perceive high levels of social support are likely to view exercise as a proactive choice in response to others’ care and expectations. Additionally, encouragement from family, friends, and peers stimulates external motivation for exercise, while the perception of being supported transforms into a sense of self-efficacy regarding the ability to persist in exercise. Under the dual motivation drive, exercise initiative is significantly enhanced [42,43].On the behavioral buffering level, older adults often face physical discomfort and loneliness during exercise, yet Perceived Social Support can effectively buffer the negative impacts of these barriers.When fatigue or mild pain occurs during exercise, the perception of support from family and friends strengthens their willpower to overcome these challenges. Moreover, when motivation to exercise is lacking due to living alone, the sense of peer presence in online communities can effectively alleviate loneliness, preventing exercise interruptions due to low mood [44,45].At the level of persistence enhancement, Perceived Social Support helps to establish a social constraint mechanism and emotional feedback loop for exercise behavior [46].The check-in system of online fitness groups and regular greetings from peers create implicit social constraints, motivating older adults to maintain regular exercise. Positive feedback from exercise results further enhances the perception of support, forming a positive feedback loop: support perception → exercise persistence → more support → easier persistence.Other studies have confirmed that Perceived Social Support is a significant predictor of physical activity levels in older adults [43,47].Based on the above theoretical and empirical analysis, the present study proposes Hypothesis 3: Perceived Social Support plays a significant mediating role in the relationship between Social Media Use of older adults and their Exercise Habits.

### 1.4 The potential chain mediation effect

Chain mediation is defined as the core mechanism in which an independent variable indirectly influences the dependent variable through the continuous transmission of multiple sequential mediators, essentially constructing a progressive path of influence from the independent variable to the dependent variable. Based on the Stress and Coping Model [48] and Social Support Theory [49], Active Coping Strategies and Perceived Social Support do not independently mediate the relationship between Social Media Use and Exercise Habits, but rather, they form a significant chain mediation effect through a progressive transmission path of individual psychological strategy activation → enhancement of social resource perception. This core chain logic aligns with the developmental pattern in which psychological adaptation precedes behavioral change in older adults, and also fits the interactive nature of social behaviors and health behaviors in the digital age. Social Media Use initially shapes Active Coping Strategies in older adults through information empowerment and social activation [6]; Active Coping Strategies facilitate older adults’ acquisition and reinforcement of support through activating proactive social behaviors and enhancing interaction quality on social media, significantly improving their Perceived Social Support [50]; ultimately, Perceived Social Support promotes the formation and maintenance of Exercise Habits by strengthening exercise motivation and buffering behavioral barriers. This chain path aligns with the core psychological development traits and behavioral logic of older adults. In the digital age, health behavior change in older adults requires not only the activation of individual-level psychological strategies but also the acquisition of external social support through social interactions, forming a progressive, continuous mediating chain that jointly mediates the effect of Social Media Use on Exercise Habits. Existing empirical studies on chain mediation have confirmed that the continuous mediation effect of coping strategies and social support can effectively predict health-promoting behaviors [51,52], providing solid methodological support for this hypothesis. Based on the above theoretical and empirical analysis, the present study proposes Hypothesis 4: Active Coping Strategies and Perceived Social Support exert a significant chain mediating effect in the relationship between Social Media Use of older adults and their Exercise Habits. Specifically, Social Media Use first influences Active Coping Strategies, which in turn affects Perceived Social Support, ultimately indirectly promoting the formation and maintenance of Exercise Habits.

**Fig 1:**
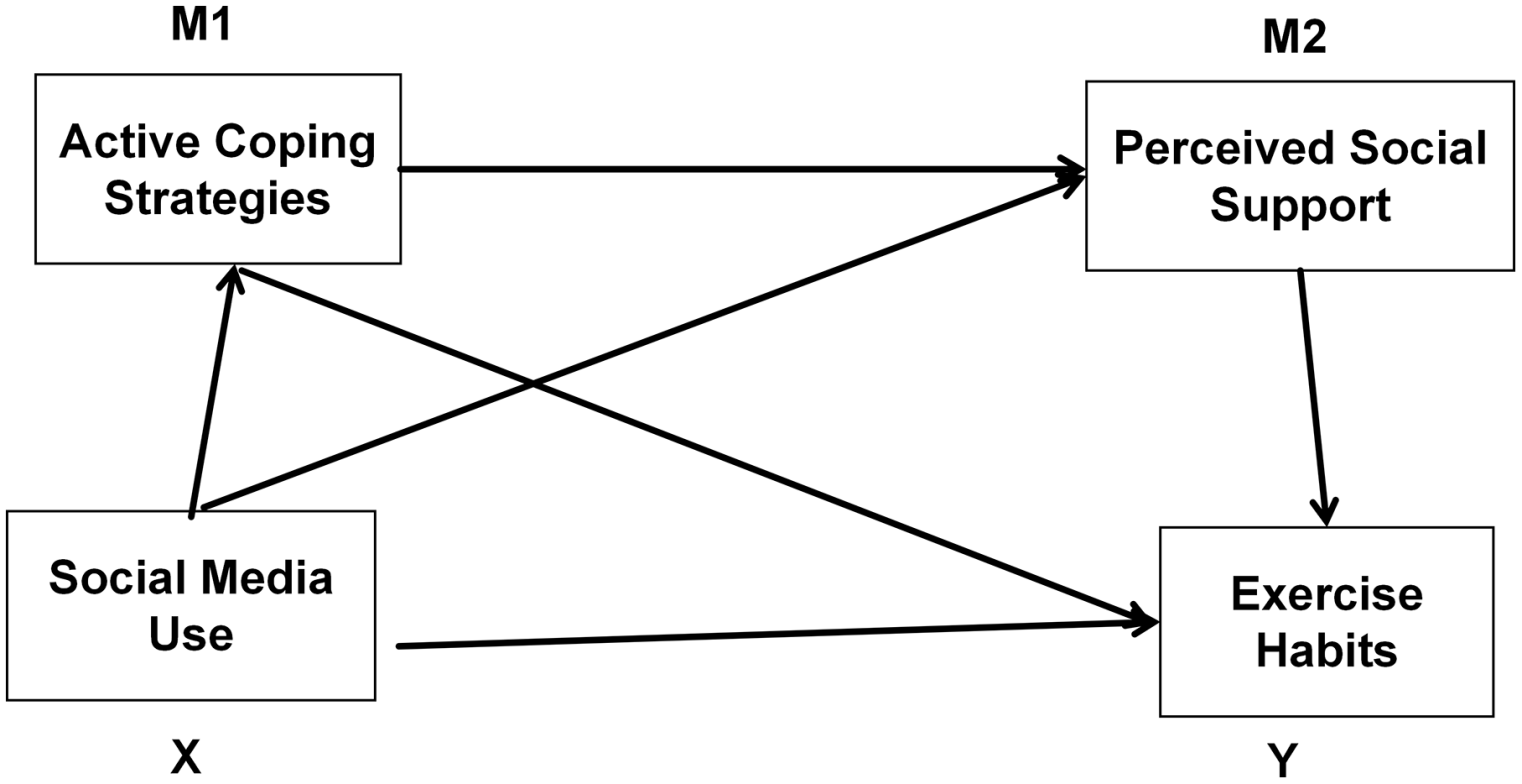
Theoretical model diagram.

Based on the above assumptions, this study proposes a theoretical model framework (see Fig 1).

## 2 Methods

### 2.1 Participants

The survey was conducted from October to December 2024 in Hunan, Hubei, and Jiangxi provinces. Ethical approval for this study was obtained from the Ethics Committee of Hunan Mechanical & Electrical Polytechnic (approval number 2024056) prior to its commencement, and the study was conducted in accordance with the Declaration of Helsinki. A purposive sampling method, balancing convenience, was employed to select older adults aged 60 and above who met the criteria of good physical health and mentally sound from urban and rural communities in these regions.

The two inclusion criteria were operationally defined and standardized assessed as follows:Good physical health: (1) Self-reported ability to complete basic daily living activities (ADLs, e.g., eating, bathing, walking) independently without assistance; (2) No acute diseases or severe physical disabilities (e.g., hemiplegia, severe joint deformity) that limit physical activity; (3) No uncontrolled chronic diseases (e.g., severe hypertension with blood pressure >180/110 mmHg, unstable angina, uncontrolled diabetes with fasting blood glucose >16.7 mmol/L) confirmed by self-report and on-site investigator verification.Mentally sound: (1) Self-reported clear consciousness and normal cognitive function; (2) On-site screening using the Mini-Mental State Examination (MMSE) (Folstein et al., 1975) with a score of ≥24/30 (the cut-off value for normal cognitive function in older adults with elementary education and above in China); (3) No history of mental illness (e.g., schizophrenia, severe depression) or cognitive impairment (e.g., dementia) reported by the participants or their family members.

The survey was assisted by local village committees, with questionnaires distributed through group briefings. In accordance with voluntary participation principles, older adults willing to participate were asked to sign informed consent forms prior to completing the questionnaire. Given the specific needs of older adult participants, enumerators read out and explained the questions during the survey, and participants filled out the questionnaires in a centralized manner. For some older adults with lower levels of education, enumerators accurately filled out the questionnaires based on the participants’ responses. A total of 1,213 questionnaires were collected, with 1,119 deemed valid, resulting in a response rate of 92.25%. The demographic characteristics of the sample are shown in Table 1.

**Table 1:** Demographic characteristics of the sample (N = 1119).

Variable	Category	*N*	*%*	*M ± SD*
Gender	Male	585	52.28	–
	Female	534	47.72	–
Age (years)	60-65	86	7.69	68.35 ± 5.21
	66-70	643	57.46	–
	71-75	314	28.06	–
	76-80	56	5.00	–
	81-85	16	1.42	–
	86 and above	4	0.35	–
Education level	Primary school and below	423	37.79	–
	Lower secondary school	451	40.31	–
	Upper secondary school / Technical college	186	16.63	–
	Higher education and above	59	5.27	–
Marital status	Married / Living with a partner	926	82.75	–
	Widowed	158	14.12	–
	Divorced / Unmarried	35	3.13	–
Type of residence	Living with family	783	69.98	–
	Living alone	236	21.09	–
	Care home	100	8.93	–
Prevalence of chronic diseases	No chronic conditions	215	19.22	–
	1 chronic condition	438	39.14	–
	2 or more chronic conditions	466	41.64	–

### 2.2 Measurements

#### 2.2.1 Social media use scale.

The questionnaire was designed based on Ellison’s framework [53], with the original reference to “Facebook” replaced by “social media,” and the developer reported a Cronbach’s α of  .83 for the original version.The original scale was translated and adapted to fit the cultural context and linguistic characteristics of China, ensuring that the meaning remained unchanged. The final Chinese version of the scale closely aligns with the original items and scoring system. The scale includes eight items, such as the number of friends on your social media, with items 3–8 being rated on a 5-point Likert scale, ranging from “Strongly Disagree” (1) to “Strongly Agree” (5). The scores were standardized, and the final score was used to determine the intensity of Social Media Use, with higher scores indicating greater usage intensity. In the present study, the Cronbach’s α for the adapted Social Media Use Scale was .87, indicating good internal consistency reliability in the target sample.

#### 2.2.2 Exercise habits scale.

The Exercise Habits Scale, developed based on the framework proposed by Weng Mengqian [54],with the original scale reporting a total Cronbach’s α of .94 and subscale α values ranging from .91,has been validated in the Chinese population [55].The scale comprises two dimensions, namely Repetitiveness (Items 1–7) and Willingness (Items 8–14), with a total of 14 items. Each item is rated on a 5-point Likert scale, ranging from “Strongly Disagree” (1) to “Strongly Agree” (5), with higher total scores indicating better quality. In the present study, the Cronbach’s α for the total Exercise Habits Scale was .97, and the α values for the repetitiveness and volition subscales were .91 and .94, respectively, indicating excellent internal consistency reliability in the older adult sample.

#### 2.2.3 Active coping strategies.

The Simplified Coping Style Questionnaire, developed by Jie Yaning [56], consists of 20 items, with two dimensions: active coping and passive coping. Items 1–12 measure active coping, and this study focuses on the active dimension of the first 12 items,with the original scale reporting a Cronbach’s α of .89 for the active coping dimension.A multi-level scoring system was used for each item, with response options ranging from “Not Used” (1) to “Frequently Used” (4),respectively. In the present study, the Cronbach’s α for the Active Coping Strategies Scale was .93, indicating excellent internal consistency reliability.

#### 2.2.4 Perceived social support scale.

The Perceived Social Support Scale, developed by Zimet [57], was used to assess the participants’ social support status,with the original scale reporting a Cronbach’s α of .88.The original scale was translated and adapted to fit the cultural context and linguistic features of China, ensuring that its meaning remained consistent. The scale consists of 12 items, each rated on a 1 (strongly disagree) to 7 (strongly agree) Likert scale. The total score is obtained by summing the scores of the 12 items, with higher scores indicating higher Perceived Social Support. A total score of ≥61 indicates high social support, ≤ 36 indicates low social support, and scores between 37 and 60 indicate moderate social support.In the present study, the Cronbach’s α for the adapted Perceived Social Support Scale was .97, indicating excellent internal consistency reliability.

### 2.3 Statistical analysis

Descriptive statistics and correlation analysis were conducted using SPSS version 26.0, while the PROCESS macro was employed to test for mediation effects. The results of the Harman’s single-factor test revealed that 13 factors with eigenvalues greater than 1 were extracted. The first factor accounted for 45.982% of the total variance, which is below the 50.00% threshold, indicating that common method bias was not a significant concern in this study [58].

## 3 Research findings

### 3.1 Descriptive statistics and correlation analysis (N=1119）

A correlation analysis was conducted on Social Media Use, Perceived Social Support, Active Coping Strategies, and Exercise Habits in the older adultspopulation. The results are shown in Table 2.

**Table 2:** Table of descriptive statistics and correlation analysis(N = 1119).

Variable	*M*	*SD*				
Social Media Use	22.99	6.69	–			
Perceived Social Support	60.77	12.45	.42***	–		
Active Coping Strategies	36.15	6.10	.46***	.64***	–	
Exercise Habits	49.96	10.87	.60***	.60***	.60***	–

Note: ** p < 0.01 *** p < 0.001, Normality was examined using the Shapiro-Wilk test. All variables showed normal distributions (p > .05).

Social Media Use was significantly positively correlated with Perceived Social Support, Active Coping Strategies, and Exercise Habits, with notable differences in the strength of the correlations. Perceived Social Support was strongly positively correlated with Active Coping Strategies and Exercise Habits, highlighting its significant supportive role in both behavioral and psychological capacities. Active Coping Strategies were significantly positively correlated with Exercise Habits. The significant correlations between the variables were consistent with the research hypotheses, providing a necessary foundation for subsequent model studies.

### 3.2 Test for mediation effect

Using Social Media Use as the independent variable, Exercise Habits as the dependent variable, and Active Coping Strategies and Perceived Social Support as mediators, the chain mediating model was tested using PROCESS Model 6, while controlling for demographic variables such as gender. The standardized regression coefficients for all paths were highly significant *(p < 0.001)*. The model demonstrated good overall explanatory power. The specific path results are shown in Table 3:

**Table 3:** Regression analysis between variables (N = 1119).

Variable	Exercise Habits			Active Coping Strategies			Perceived Social Support			Exercise Habits		
	*β*	*SE*	*t*	*β*	*SE*	*t*	*β*	*SE*	*t*	*β*	*SE*	*t*
Social Media Use	.60	.04	25.12***	.46	0.02	17.39***	.16	.05	6.22***	.37	.04	15.73***
Active Coping Strategies							.56	.05	21.99***	.25	.05	8.95***
Perceived Social Support										.29	.02	10.87***
*R 2*	.36				.21		.43			.54		
*F*	631.15***			302.52***			412.19***			438.28***		

Note: * p < 0.05 ** p < 0.01***p < 0.001, Normality was examined using the Shapiro-Wilk test. All variables showed normal distributions (p > .05).

The total effect of Social Media Use on Exercise Habits was significant, explaining 36.1% of the variance in Exercise Habits. The predictive effect of Social Media Use on Active Coping Strategies was significant, explaining 21.3% of the variance in Active Coping Strategies. The predictive effect of Social Media Use on Perceived Social Support was also significant. The predictive effect of Active Coping Strategies on Perceived Social Support was significant, explaining 42.5% of the variance in Perceived Social Support. The predictive effect of Active Coping Strategies on Exercise Habits was significant, and the predictive effect of Perceived Social Support on Exercise Habits was also significant. The direct effect of Social Media Use on Exercise Habits remained significant after controlling for the mediating variables.

To test thechain mediating effect of Active Coping Strategies (M1) and Perceived Social Support (M2) in the relationship between Social Media Use (X) and Exercise Habits (Y), PROCESS macro (Model 6) was used, and the bias-corrected non-parametric percentile bootstrap method (with 5000 resamples) was applied to calculate the 95% confidence interval (CI), to determine the significance of the effect (an effect is considered significant if the confidence interval does not include 0). The specific results of the chain mediating effect test are shown in Table 4.

**Table 4:** Chain mediation effect analysis.

*Effect*	Influence pathway	*Effect*	*SE*	*LLCI*	*ULCI*
Total Effect	Social Media Use⇒Exercise Habits	.98	.04	.90	1.05
Direct effect	Social Media Use⇒Exercise Habits	.60	.04	.52	.67
Total Indirect Effect	Social Media Use=>Exercise Habits	.38	.02	.20	.27
Indirect Effect	X=>M1=>Y	.18	.01	.09	.15
	X=>M2=>Y	.08	.01	.03	.06
	X=>M1=>M2=>Y	.12	.01	.06	.10

The results indicate that Social Media Use has a significant total effect on the Exercise Habits of older adults, suggesting that, in the absence of mediating variables, the level of Social Media Use positively predicts the development and maintenance of Exercise Habits among the elderly. When both Active Coping Strategies and Perceived Social Support were included as mediating variables, the direct effect of Social Media Use on Exercise Habits remained significant, indicating that Social Media Use not only indirectly influences Exercise Habits through the mediating pathways but also has a direct positive effect on Exercise Habits. The core test results of the chain-mediated effect show that Social Media Use has a significant total indirect effect on Exercise Habits through Active Coping Strategies and Perceived Social Support, accounting for 39.10% of the total effect. This suggests that the mediating pathways are a crucial mechanism through which Social Media Use affects Exercise Habits.

The total indirect effect was further decomposed, and the effects of the three specific mediation paths were all significant (95% CI did not include 0). The first direct mediation path (X → M1 → Y): the effect of Active Coping Strategies as a mediator was significant, and this path accounted for 48.17% of the total indirect effect. This suggests that Social Media Use positively shapes older adults’ Active Coping Strategies, thereby promoting the development of their Exercise Habits, which supports Hypothesis 2. The second direct mediation path (X → M2 → Y): the effect of Perceived Social Support as a mediator was significant, and it accounted for 19.63% of the total indirect effect. This finding confirms that Social Media Use can indirectly promote the maintenance of Exercise Habits by expanding the social support network of older adults and enhancing their perception of emotional, instrumental, and informational support, which supports Hypothesis 3. The chain mediating path (X → M1 → M2 → Y): the chain mediating effect of Active Coping Strategies and Perceived Social Support was significant, and it accounted for 32.20% of the total indirect effect. This result suggests that Social Media Use first positively predicts Active Coping Strategies, and Active Coping Strategies further strengthen older adults’ perception of social support by activating their proactive social behaviors and improving the quality of online interactions, ultimately promoting the formation and consolidation of Exercise Habits, fully supporting Hypothesis 4.

## 4 Discussion

### 4.1 Social media use has a positive direct effect on exercise habits

This study found that Social Media Use is significantly positively associated with Exercise Habits in the elderly. This finding not only supports the core expectation of Hypothesis 1 but also aligns with Social Cognitive Theory and the Stress and Coping Model. After controlling for the chain mediation effects of Active Coping Strategies and Perceived Social Support, Social Media Use remains significantly positively associated with the development and maintenance of Exercise Habits in the elderly, and the cross-sectional data suggests a potential predictive relationship. Social Media Use can directly and positively predict the Exercise Habits of the elderly. Information, social interaction, and motivational resources provided by digital platforms can reduce barriers to exercise [59]. Elderly individuals can access age-appropriate and readily available exercise guidance via social media, thereby enhancing self-efficacy and the sustainability of their behaviors.According to Social Cognitive Theory [60], the direct positive effect of Social Media Use on Exercise Habits in the older adultscan be explained through three core mechanisms, all of which align with the digital usage characteristics and inherent health behavior patterns of the older adultspopulation.First, the immediate empowerment path through information acquisition.Social Media Use directly drives the conversion of exercise behavior by reducing the decision-making and execution costs associated with exercise [61].One of the main barriers to exercise behavior in the older adultspopulation is the information asymmetry related to exercise. Social media, with its convenience, precision, and visual characteristics, directly bridges this information gap.A large amount of low-intensity exercise tutorials and chronic disease rehabilitation exercise guidelines adapted for the older adultspopulation on short video platforms lowers the threshold for information comprehension through intuitive video formats.At the same time, the algorithmic recommendation system can push personalized content based on user preferences, allowing the older adultsto access highly relevant exercise information without active searching, significantly reducing the initiation cost of exercise behavior.An empirical study on older adultspopulations in Chinese cities found that the accessibility and applicability of health information on social media directly predict exercise frequency [62], which is highly consistent with the results of this study—when the barrier to accessing exercise information is minimized, older adults’ exercise decisions and execution become more immediate, leading to a direct behavior promotion effect.Second, the behavioral activation path through social interaction.Exercise behavior in the older adultspopulation exhibits a significant social dependence, with traditional offline social interactions constrained by time and space. In contrast, the online-offline interactive function of social media directly activates the reinforcement cycle of exercise behavior.For example, features such as WeChat Sports step counting and daily check-ins in fitness groups directly strengthen exercise behavior through peer comparison and group identity, without the need for complex psychological intermediaries.Some online fitness groups organize offline group exercise activities, further converting online social interactions into offline exercise behavior, thereby achieving a direct transmission effect from digital social interaction to exercise behavior activation.The immediate interactive features of social media are directly related to exercise adherence [63,64], and this mechanism is further validated in the present study.The immediacy and convenience of social interaction enable it to bypass psychological intermediary variables and directly affect the maintenance of Exercise Habits.Third, the immediate motivational path through role model demonstration. The demonstrative effect of senior fitness influencers on social media has a direct stimulating effect on exercise behavior. Compared to traditional media, social media is characterized by stronger interactivity and authenticity. Exercise routines shared by senior creators not only provide behavioral examples to emulate, but also convey positive cognitive beliefs. This role model effect among peers directly stimulates the exercise approach motivation in the older adultspopulation. The exercise cases of peers on social media have a significantly stronger direct predictive power on the exercise behavior of the older adultscompared to younger populations [59,65]. This aligns closely with the findings of the present study, which shows that the older adultsexhibit a stronger sense of identification with peer role models, and the behavioral conversion efficiency of role model demonstration is higher, resulting in a direct facilitative effect of Social Media Use on Exercise Habits. Unlike some studies on middle-aged and young populations which emphasize the indirect effect of Social Media Use through psychological mediators [25,52], this study finds that the direct effect of Social Media Use is more prominent in the older adultspopulation. This discrepancy may arise from the elderly’s greater focus on the practical functions of digital media, resulting in a shorter behavioral conversion chain, highlighting the value of this study in refining the exploration of digital health behavior mechanisms across different age groups.

### 4.2 Active coping strategies mediate the positive associational relationship between social media use and exercise habits

The results indicate that the independent mediating effect of Active Coping Strategies between Social Media Use among older adults and Exercise Habits is significant and positive, which validates Hypothesis 2. Consistent with the theoretical expectations proposed based on the Stress and Coping Model, Active Coping Strategies exert a mediating effect between Social Media Use and Exercise Habits. The findings of this study demonstrate that social media helps improve problem-solving efficacy and active participation levels, thereby facilitating the formation of stable Exercise Habits. This result is also consistent with the Conservation of Resources Theory: digital resources help older adults acquire and maintain psychological resources, thereby enhancing their coping adaptability.The core stressors faced by older adults in exercise behavior are essentially psychological stressors that require active coping [66].Empirical studies have confirmed that digital media use significantly enhances psychological resilience and positive emotions in older adults [30,67], which are the core foundations of Active Coping Strategies, aligning with the transmission logic of Social Media Use → Active Coping Strategies in this study.Older adults with a tendency for active coping are more likely to view exercise as a proactive solution to improve health and delay aging, rather than as a task they are compelled to complete [68,69].This intrinsic motivation can effectively enhance exercise engagement, preventing abandonment due to short-term difficulties.For example, when faced with muscle soreness after exercise, active copers interpret it as a normal process of physical adaptation, rather than as a signal of harmful effects, thereby sustaining their exercise motivation.A substantial amount of exercise knowledge tailored to older adults is shared through social media in a visual and simplified format, helping them objectively recognize the feasibility and practicability of exercise, reducing negative perceptions such as fear of injury or lack of knowledge, and strengthening positive beliefs that problems can be solved, thereby enhancing problem-solving ability [70,71].For instance, older adults can effectively cope with the constraints of fragmented time by learning short, frequent exercise methods, developing adaptive strategies that involve adjustments rather than abandoning exercise.The problem-solving ability within Active Coping Strategies help older adults overcome specific barriers in exercise.Choosing low-intensity exercises such as Tai Chi or Eight-Section Brocade for joint pain, joining online fitness groups for collective check-ins due to living alone, and creating a daily exercise schedule of 3 sessions, each lasting 10 minutes, to address fragmented time—these specific problem-solving ability can effectively reduce the difficulty of executing exercise behaviors and enhance their sustainability [72,73]. The findings of this study are consistent with those of Niemelä R et al. [74] and Li et al. [75] which confirm that digital media use can indirectly promote health behaviors in older adults by enhancing active coping tendencies, thereby reinforcing the study’s conclusions. This also aligns with previous research showing a positive correlation between coping strategies and physical activity in older adults [19,76], validating the universal value of Active Coping Strategies in older adultshealth behaviors.The mediating mechanisms identified in this study illuminate how digital behaviors are transformed into health-promoting actions, thereby extending the theoretical framework of stress and coping in the elderly.

### 4.3 Perceived social support mediates the positive associational relationship between social media use and exercise habits

The results of this study demonstrate that Perceived Social Support significantly and positively mediates the relationship between Social Media Use of Older Adults and Exercise Habits, thus validating Hypothesis 3. This suggests that Social Media Use can indirectly promote the development and maintenance of Exercise Habits by activating the perception of social resources in older adults. This reveals the key socio-psychological mechanism through which social media influences exercise behavior in older adults: digital media not only shapes internal psychological strategies but also restructures external social support networks, indirectly empowering health behaviors by enhancing perceived support.Empirical studies have confirmed [77,78]that the frequency of Social Media Use in older adults is significantly positively correlated with Perceived Social Support, and digital social participation effectively expands the size and heterogeneity of their social support networks, thereby enhancing the effectiveness of perceived support. This is consistent with the mediation logic of Social Media Use → Perceived Social Support in the current study. According to the main effect model of social support theory, high levels of Perceived Social Support can directly enhance an individual’s motivation for health behaviors. On one hand, older adults who perceive concern and expectations from family, friends, and communities regarding their health are likely to view exercise as an external responsibility to respond to others’ care, thus forming extrinsic motivation [42]; on the other hand, the perception of being supported can translate into a sense of self-efficacy in maintaining exercise, thereby strengthening intrinsic motivation [79].The synergistic drive of dual motivation significantly enhances the initiative and conscientiousness of exercise. For example, older adults are more likely to develop a regular exercise habit because they do not want to disappoint the encouragement of fitness group peers (extrinsic motivation) and believe they can persist with the support of their peers (intrinsic motivation). Based on the buffering model of social support theory, Perceived Social Support can buffer the interference of negative factors during exercise. Common barriers to exercise behavior in older adults include physical discomfort and low mood, but the perception of social support can effectively counteract these barriers. When fatigue occurs during exercise, the perception of support from peers who cheer them on can strengthen their willpower to overcome difficulties; when lacking motivation due to living alone, the sense of presence from online community peers can alleviate loneliness and prevent exercise interruptions caused by low mood [43,80].The results of this study are consistent with those of Wu et al. [81] and Sheng et al. [26], both confirming the positive role of online Perceived Social Support in promoting exercise behavior in older adults. This further reinforces the conclusion that social support is a key protective factor for health behaviors in older adults.

### 4.4 Active coping strategies and perceived social support exert a chain-mediated effect between social media usage and exercise habits

The results of this study show that the chain-mediated effect is significant and positive, thus confirming Hypothesis 4. This suggests that social media first shapes older adults’ Active Coping Strategies through information empowerment and social activation, and Active Coping Strategies further enhance their Perceived Social Support by activating proactive social behaviors, ultimately facilitating the formation and maintenance of Exercise Habits. The existence of this chain-mediated effect reveals the resource-strategy integration mechanism through which social media influences older adults’ exercise behavior, complementing the two separate mediation paths and collectively forming a multidimensional and multilayered comprehensive impact mechanism.Social media, through mechanisms such as cross-temporal and spatial connections, the construction of interest-based groups, and the provision of diverse types of support [14,20], has broken the spatial-temporal limitations and monotony of traditional social support for older adults, allowing them to access continuous and diverse forms of emotional, informational, and instrumental support. The core value of this digital social support lies in the enhanced resource accessibility—older adults can conveniently access exercise-related support resources without relying on offline social interactions, thus strengthening their subjective perception that their exercise behavior is supported and that assistance is available when facing difficulties [82,83].It is worth noting that the social resources accumulated through social media are not passive external gifts but rather provide a psychological safety net for the subsequent formation of Active Coping Strategies, giving older adults the external confidence to actively cope with exercise-related stress.High levels of Perceived Social Support can reduce older adults’ perception of exercise-related stress [49].When older adults face stressors such as exercise difficulties due to physical decline or feelings of helplessness caused by lack of professional guidance, their subjective perception of social support enables them to interpret these stressors as challenges that can be alleviated through external support rather than insurmountable crises, thereby reducing the tendency for passive avoidance and creating the psychological conditions for activating Active Coping Strategies [84,85].Under the psychological resources supported by the transformation of Perceived Social Support, the promoting effect of Active Coping Strategies on Exercise Habits becomes more stable.On one hand, Active Coping Strategies reduce the execution difficulty of exercise behavior through cognitive restructuring, problem-solving ability, and other strategies; on the other hand, the sense of being supported brought by Perceived Social Support strengthens the continuity of active coping—when older adults adopt active strategies to overcome exercise difficulties, they not only receive positive feedback on the exercise outcomes but also feel the recognition from their support network, forming a virtuous cycle of support → active coping → behavioral success → more support [43,80], thus facilitating the transformation of exercise behavior from occasional participation to a stable habit.

### 4.5 Limitations

This study has several limitations. First, this study adopts a cross-sectional design, which only allows for the examination of the relationships between Social Media Use, Exercise Habits, Active Coping Strategies, and Perceived Social Support. It does not explore in depth the individual’s Exercise Habits and Social Media Use patterns, nor the evolving effects on self-control abilities and body image. Future studies are recommended to adopt stratified random sampling or multi-stage probability sampling, incorporating a more representative sample, particularly groups from different socioeconomic statuses and cultural backgrounds, to test the cross-group generalizability of the research model. Second, the data in this study were collected at a single time point, and future research could collect data at multiple time points, combining objective data with self-reports, and employ a mixed-methods design to enhance measurement validity and reduce common method bias. Third, this study only considered the effects of Active Coping Strategies and Perceived Social Support, future studies should include additional relevant mediating variables, to provide a more comprehensive understanding of the interactive mechanisms within the model. Fourth, this study treated Social Media Use as a single variable without distinguishing between content types. Different types of social media content may have heterogeneous effects on Exercise Habits—for example, exercise-related content may directly promote exercise behavior, while entertainment content may have a time-substitution effect. Future studies should adopt content analysis or experimental designs to clarify the differential impacts of various content types.Finally,This study used convenience sampling, resulting in a higher proportion of participants aged 66–70, living with family, and with a lower education level, along with gender imbalance. Future research should consider adopting stratified random sampling to increase the representation of older, single, and lower-educated groups, and include gender as a variable in the analysis, thereby enhancing the generalizability of the results.

## 5 Conclusion and recommendations

This study focuses on the relationship between Social Media Use and Exercise Habits, verifying the serial mediating effect of Active Coping Strategies and Perceived Social Support, providing a new perspective for understanding the formation mechanisms of health behaviors in the digital age. The influence of Social Media Use on Exercise Habits does not occur directly but is transmitted through a dual psychological pathway of Active Coping Strategies leading to Perceived Social Support. This study extends the application boundaries of social support theory in the digital age. As a novel digital environmental stimulus, social media can simultaneously activate individual psychological strategies and external social resources, enriching the theoretical pathway of environment-behavior interaction (extending from traditional offline environments to digital environments). An integrated model was developed for the direct effects, dual separate mediations, and chain mediating of older adultshealth behaviors. By quantifying the contribution ratios of each pathway, the relative importance of different mechanisms was clarified, addressing the gap in previous research regarding the exploration of multiple mechanisms underlying digital health behaviors in the elderly.

Based on the findings of this study and the digital usage characteristics and health behavior patterns of the older adultspopulation, the following recommendations are proposed:Policy level. Strengthen institutional guarantees for the integration of digital aging adaptation and older adultshealth, incorporating digital skills training and health behavior promotion into relevant policy guidelines. Tax incentives and financial subsidies should be provided to support the development of senior-friendly digital health products, and a cross-departmental collaborative mechanism involving the Ministry of Civil Affairs, National Health Commission, and Cyberspace Administration should be established. Additionally, guidelines for promoting older adultsdigital health behaviors should be formulated, creating a unified policy effort.Social media platform level: Optimize the provision of resources, psychological empowerment, and interactive support through age-friendly designs. Algorithms should be leveraged to precisely deliver low-intensity, visually adapted exercise content and coping strategy guidance for the elderly. Additionally, functions such as recommendations for senior fitness communities, exercise check-in reminders, and reducing social interaction barriers should be incorporated. The development of role models and interactive incentive mechanisms should be based on examples of silver-haired fitness enthusiasts.Community and family collaboration level. Develop an intervention model integrating online support and offline practice. Communities should take the lead in organizing digital fitness group training and offline collective exercise activities. The involvement of children in encouraging Social Media Use and exercise among the older adultsshould be promoted. Online groups can be used to collect needs, and targeted guidance can be provided in collaboration with professionals. A system for exercise monitoring and feedback should be established.Individual level for the elderly. Actively participate in digital skills training at community centers or senior universities, with a focus on improving health information retrieval and online fitness interaction skills. The older adults should actively join fitness-related peer groups, leveraging peer support and role model demonstration to foster positive coping tendencies. Personalized exercise plans should be developed based on individual health conditions, and digital resources should be flexibly utilized to overcome exercise barriers.

## Supporting information

S1 Data Supporting data for this paper. (XLSX)
